# Supplementary material for: The gold content of mafic to felsic potassic magmas
Source: Nat Commun. 2024 Aug 14;15:6988. doi: 10.1038/s41467-024-51405-7 (PMC11324659; doi:10.1038/s41467-024-51405-7)
Supplement: Supplementary file 1 — Supplementary Information [file 41467_2024_51405_MOESM1_ESM.pdf]

The gold content of mafic to felsic potassic magmas

Jia Chang[1, *], Andreas Audétat[1], Thomas Pettke[2]

[1]Bavarian Geoinstitute, University of Bayreuth, Bayreuth 95440, Germany

[2]Institute of Geological Sciences, University of Bern, Bern 3012, Switzerland

*Email: Jia.Chang@uni-bayreuth.de

This file contains three Supplementary Figures:

Supplementary Fig. 1

Supplementary Fig. 2

Supplementary Fig. 3

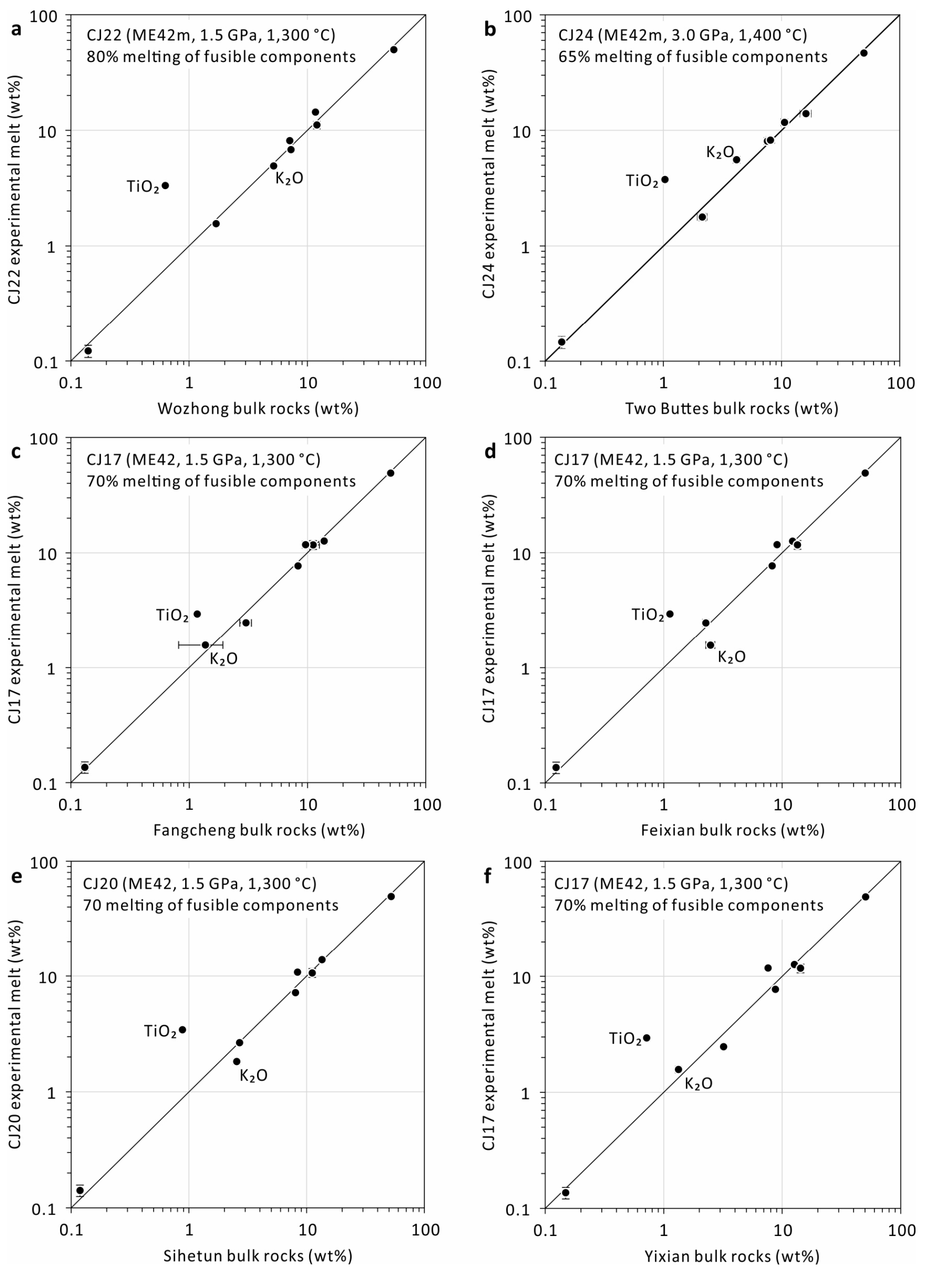

Supplementary Fig. 1 Major element oxide contents of natural high-Mg potassic mafic rocks, compared to those of experimentally produced partial melts. (a) Wozhong bulk rocks vs. CJ22 experimental melt, (b) Two Buttes vs. CJ24, (c) Fangcheng vs. CJ17, (d) Feixian vs. CJ17, (e) Sihetun vs. CJ20, (f) Yixian vs. CJ17. Note the generally good match of most elements, except for TiO_2. The latter is due to extraordinarily high TiO_2 contents of the amphibole and phlogopite in the starting materials of this study. See Supplementary Fig. 2 for the definition of fusible components.

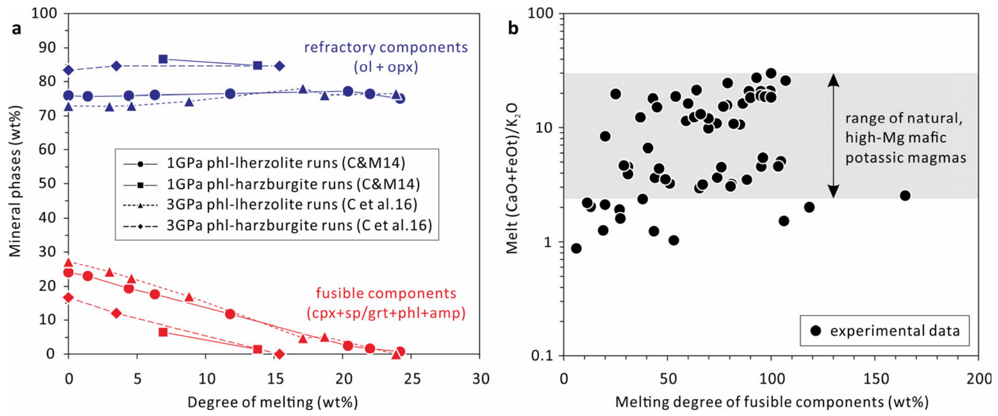

Supplementary Fig. 2 Experimental constraints on the degree of partial melting of metasomatic mantle components. (a) The degree of partial melting of phl ± amp-bearing peridotites vs. the abundances of fusible and refractory mantle components. All the data are taken from previous experimental studies[1,2]. Note that the total abundance of refractory components remains relatively constant, though the ol/opx ratio increases significantly (not shown) to buffer the composition of the partial melts. (b) Experimentally constrained melting degrees of fusible components vs. the $(CaO+FeOt)/K_2O$ ratio of the silicate melts. The dataset includes all partial melting experiments on natural mantle phl- and/or amp-bearing peridotites and pure metasomatic veins that are available to date[1–3]. Melting degrees >100% indicate that not only the fusible components were melted, but also part of the refractory components. The range of $(CaO+FeOt)/K_2O$ ratios in natural magmas is based on a global dataset[4,5, this study]. The lack of compositional overlap between experimental and natural melt compositions at very low and very high melting degrees suggest that the natural alkaline magmas formed at melting degrees in the range of ~20–100%. Abbreviations: amp, amphibole; cpx, clinopyroxene; grt, garnet; ol, olivine; opx, orthopyroxene; phl, phlogopite; sp, spinel.

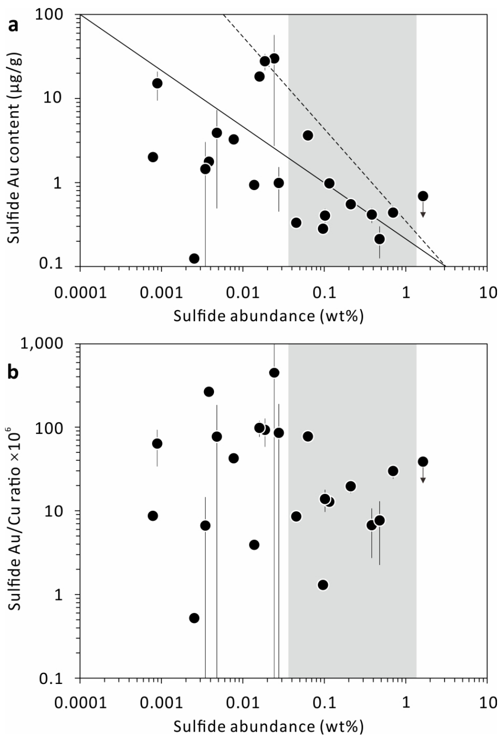

Supplementary Fig. 3 Measured Au content and Au/Cu ratio of sulfides vs. observed sulfide abundance in mantle metasomatic veins. The grey shaded areas reflect the range in sulfide abundances expected to have been present in the metasomatic veins that produced the mafic potassic magmas investigated in this study, calculated based on the S content of the mafic potassic magmas (0.07–0.42 wt% S), a sulfide S content of 36.5 wt%, and melting degrees of 20−100% in the metasomatic veins (note that the sulfides were exhausted during the partial melting). In panel (a), the solid line stands for moderate sulfide Au contents as a function of sulfide abundances, whereas the dash line stands for very high sulfide Au contents. Based on the sulfide abundances and sulfide Au contents covered by the grey area, the Au contents in the primitive potassic melts were then calculated using the same range of melting degrees as above, returning 1.2–6.6 ng/g for the case of moderate sulfide Au contents, and 3.5–25 ng/g for the case of very high sulfide Au contents. The melts would contain 20–25 ng/g only in the most extreme, probably unrealistic, scenario of a metasomatic vein that contains the very Au-rich sulfides and undergoes only 20% melting. The Au/Cu ratios of

the primitive potassic melts should be the same as that of the mantle sulfides as shown in panel (b), because the sulfides are completed dissolved upon partial mantle melting.

References

1. Condamine, P. & Médard, E. Experimental melting of phlogopite-bearing mantle at 1 GPa: Implications for potassic magmatism. *Earth Planet. Sci. Lett.* **397**, 80–92 (2014).
2. Condamine, P., Médard, E. & Devidal, J. L. Experimental melting of phlogopite-peridotite in the garnet stability field. *Contrib. Min. Petrol.* **171**, 95 (2016).
3. Lloyd, F. E., Arima, M. & Edgar, A. D. Partial melting of a phlogopite-clinopyroxenite nodule from south-west Uganda: an experimental study bearing on the origin of highly potassic continental rift volcanics. *Contrib. Min. Petrol.* **91**, 321–329 (1985).
4. Pilet, S., Baker, M. B. & Stolper, E. M. Metasomatized lithosphere and the origin of alkaline lavas. *Science* **320**, 916–919 (2008).
5. Schmidt, M. W. & Jagoutz, O. The global systematics of primitive arc melts. *G-Cubed* **18**, 2817–2854 (2017).
