# Supplementary material for: The gold content of mafic to felsic potassic magmas
Source: Nat Commun. 2024 Aug 14;15:6988. doi: 10.1038/s41467-024-51405-7 (PMC11324659; doi:10.1038/s41467-024-51405-7)
Supplement: Supplementary file 3 — Description Of Additional Supplementary File [file 41467_2024_51405_MOESM3_ESM.pdf]

Description of Additional supplementary files

File name: Supplementary data 1.

Description: Petrography of the studied mafic potassic rocks

File name: Supplementary data 2.

Description: Compositions of individual melt inclusions analyzed by LA-ICP-MS in mafic potassic rocks

File name: Supplementary data 3.

Description: Compositions of sulfide inclusions in mafic potassic rocks analyzed by LA-ICP-MS

File name: Supplementary data 4.

Description: Results of oxybarometry based on V concentrations in melt inclusion-olivine pairs analyzed by LA-ICP-MS and results of oxybarometry based on compositions of spinel inclusion-olivine pairs analyzed by EPMA

File name: Supplementary data 5.

Description: Abundance of sulfides in fusible mantle components

File name: Supplementary data 6.

Description: Compositions of sulfide inclusions in mantle rocks analyzed by LA-ICP-MS

File name: Supplementary data 7.

Description: Run conditions and product modes of mantle partial melting experiments

File name: Supplementary data 8.

Description: Compositions of experimental melts analyzed by EPMA and results of oxybarometry based on V concentrations in experimental melts and olivine analyzed by LA-ICP-MS

File name: Supplementary data 9.

Description: Compositions of melt inclusions analyzed by LA-ICP-MS in variably evolved potassic rocks from the Sanjiang region

File name: Supplementary data 10.

Description: Compositions of glassy melt inclusions (plus glass standards) analyzed by EPMA
